# Efficiency and safety of cannabinoid medical use: an analysis of discussions and observed trends on Instagram

**DOI:** 10.3389/fpubh.2024.1494018

**Published:** 2024-12-04

**Authors:** Olena Litvinova, Bikash Baral, Thomas Wochele-Thoma, Maima Matin, Nikolay T. Tzvetkov, Olga Adamska, Agnieszka Kamińska, Marcin Łapiński, Artur Stolarczyk, Atanas G. Atanasov

**Affiliations:** ^1^Department of Management, Marketing and Quality Assurance in Pharmacy, National University of Pharmacy of the Ministry of Health of Ukraine, Kharkiv, Ukraine; ^2^Ludwig Boltzmann Institute Digital Health and Patient Safety, Medical University of Vienna, Vienna, Austria; ^3^Organismal and Evolutionary Biology Research Programme, Faculty of Biological and Environmental Sciences, University of Helsinki, Helsinki, Finland; ^4^Institute of Genetics and Animal Biotechnology of the Polish Academy of Sciences, Magdalenka, Poland; ^5^Department of Biochemical Pharmacology and Drug Design, Institute of Molecular Biology “Roumen Tsanev”, Bulgarian Academy of Sciences, Sofia, Bulgaria; ^6^Faculty of Medicine, Collegium Medicum Cardinal Stefan Wyszyński University in Warsaw, Warsaw, Poland; ^7^Orthopaedic and Rehabilitation Department, Medical University of Warsaw, Warsaw, Poland

**Keywords:** cannabidiol, cannabinoid, Epidiolex, Instagram, Marinol, Nabilone, Sativex, social media

## Abstract

**Background:**

Cannabis and its derivatives show encouraging therapeutic effects in the treatment of various diseases. However, further studies are needed to better assess their efficacy and safety. A promising base for research in the field of medicine and additional pharmacovigilance is social networks, in which experience and knowledge are exchanged between researchers, doctors, and patients, as well as information about the potential risks and benefits of using drugs for medical purposes is disseminated. The aim of this study was to investigate the reported efficiency and safety of medical use of cannabinoids in patients using posts on the social media Instagram and analyze the observed trends.

**Methods:**

Social media listening platform Apify was used to collect data with hashtags as of June 4, 2024, including posts from 2023 and 2024, with some data extending into later periods, in compliance with a systematic approach to data collection. The analysis of the data obtained from the research was conducted using the RStudio platform.

**Results:**

The analysis covered 1,466 posts containing hashtags related to cannabinoids. The posts studied were categorized as follows: 33.08% focused on advertising and commercialization, 25.58% on personal experience, 21.35% on other topics, and 19.99% contained educational content. An analysis of overall content relevance found that the majority of Instagram posts (81.79%) related to cannabis and cannabinoid hashtags are relevant. Most of the Instagram posts studied were posters, followed by personal photos and videos. The analysis shows that English dominates the studied category (70.74% of posts), while German, French, Spanish, and other languages also occupy a significant place, emphasizing the importance of a multilingual approach in content analysis. It has been revealed that organizations publish a larger percentage of posts under this study, with a higher percentage of relevance. Personal experience stories receive a significant number of “likes” indicating a strong emotional connection between audience and content. Instagram discussions about cannabinoid treatment support evidence from scientific studies about their effectiveness in treating a range of diseases, such as epilepsy with Lennox–Gastaut and Dravet syndromes, multiple sclerosis, cancer, and HIV-cachexia, nausea and vomiting caused by chemotherapy. At the same time, they emphasize the need for further clinical studies to better assess safety, side effects, and optimal dosages. Advertising and commercial posts can contribute to increased cannabis use, highlighting the need to raise awareness of risks and strengthen preventive measures.

**Conclusion:**

Analysis of content on the social media Instagram can complement traditional scientific research by providing information on the real use of cannabis and its derivatives, contributing to the development of safe and effective recommendations for its use.

## Introduction

Currently, there is increasing interest in the medical use of cannabis and its derivatives, which may lead to a broader range of therapeutic options and improved patients’ quality of life when used according to prescribed instructions. For a long time, the status of cannabis as a narcotic substance has prevented its use in medical research and the treatment of various diseases. However, with legalization in a number of US states and in many other countries, interest in its medical use has increased significantly over the past decade (1–5).

Cannabis contains a variety of biologically active substances, including cannabinoids, terpenoids, flavonoids, and alkaloids. More than 70 cannabinoids are known, but the most studied are three of them: cannabinol (CBN), cannabidiol (CBD), and delta-9-tetrahydrocannabinol (THC). Their main target is cannabinoid receptors of types 1 and 2 found in the human body (type 1: central nervous system (hypocampus, cerebellum, hypothalamus, prefrontal cortex), smooth muscle, myocardium, adipocytes, neurons of the autonomic nervous system; type 2: macrophages, B lymphocytes, spleen, tonsils, bone marrow, vascular endothelium) (6).

Studies by various authors demonstrate the importance of cannabinoid receptors in physiological processes and their therapeutic potential for a variety of diseases. In a study by Zhang et al., the authors found that cannabinoid CB2 receptors are expressed in midbrain dopaminergic neurons. Their activation inhibits the neuronal activity of dopamine and the response to intravenous cocaine administration, which may be significant for psychiatric disorders (7). Evidence for the involvement of genetic cannabinoid receptor polymorphisms in a variety of diseases, including neurological and psychiatric disorders, has been summarized by Vasileiou et al. (8). In the work of Mechoulam et al., CB1 and CB2 receptors have been shown to interact with anandamide and 2-arachidonyl-glycerol, affecting movement coordination and immune functions, expanding the understanding of their role in neurophysiology (9). Studies suggest that cannabinoids, when interacting with CB1 and CB2 receptors, exhibit analgesic and anti-inflammatory effects, as highlighted by the work of Miranda-Cortés et al. and Starowicz et al. (10, 11). These properties make cannabinoids promising for multimodal pain management as well as management of conditions such as seizures, epilepsy, dermatitis, degenerative myelopathies, asthma, diabetes, and glaucoma in humans and animals. Research is underway to separate their psychoactive effects from analgesic properties. The endogenous cannabinoid system, as emphasized by Rodríguez De Fonseca, is a universal signaling system opening new therapeutic avenues for the treatment of various pathologies, including pain, obesity, neurological diseases (such as multiple sclerosis), emotional disorders (including anxiety), and psychiatric disorders such as drug dependence and alcoholism (12). A study conducted by Galiazzo et al. demonstrated the presence of cannabinoid receptors in the gastrointestinal tract of dogs, which may be useful for the therapy of inflammatory bowel diseases (13). Walter et al., reported regulatory role of the endocannabinoid system in neuroinflammation. The authors note that cannabinoids improve MS symptoms in rodent models, opening prospects for the development of new therapies (14).

THC has properties that can cause euphoria and relaxation, as well as enhance sensory perception, making it popular for recreational use. However, CBD, although a component of hemp, does not have such pronounced psychoactive effects. It, conversely, can mitigate the psychoactive properties of THC. In addition, CBD has antioxidant, anti-inflammatory, and neuroprotective properties, which makes it valuable in medical practice. When ingested, they have low bioavailability compared to inhalation methods. There is also a class of synthetic cannabinoids (dronabilone, nabilone, etc.) as well as endogenous cannabinoids (6, 15–19).

Cannabinoids have been extensively investigated in the context of their efficacy in the treatment of pain, spasticity in multiple sclerosis and spinal injury, nausea and vomiting induced by chemotherapy, and in the treatment of anorexia associated with HIV and cancer cachexia, epilepsy, Dravet syndrome, Lennox–Gastaut syndrome (20–26).

The endocannabinoid system also plays an important role in maintaining gastrointestinal (GI) homeostasis. There is now abundant evidence that cannabis and cannabinoids have anti-inflammatory and antinociceptive effects. This means that many patients with GI pathologies may benefit from their use. Results from several studies support the efficacy of cannabis or cannabinoids in patients with functional GI disorders such as gastroparesis and irritable bowel syndrome, as well as inflammatory bowel disease, non-alcoholic fatty liver disease (NAFLD), and obesity (27–29).

However, the potential use of cannabinoids in pharmacology is limited by the presence of narcotic activity, which sometimes exceeds the useful pharmacological effect. The biological activity of cannabinoids is directly related to their effects on cannabinoid receptors of the first and second types. The pharmacological activity of cannabinoids can be predicted depending on the degree of their affinity for certain types of receptors (30).

There are now numerous publications on the possible risks of medical cannabis use. However, in a number of countries (Australia, Germany, Great Britain, Denmark, Canada, the USA, Switzerland, and others), cannabioid-based drugs are registered and approved for medical use (31).

Sativex (nabiximols) is a drug developed for the treatment of pain syndrome, multiple sclerosis, and cancer. It contains a combination of two major cannabinoids, delta-9-tetrahydrocannabinol (THC) and cannabidiol (CBD), and is presented as an oral spray (32). One dose spray (100 μL) contains 2.5 mg CBD and 2.7 mg THC. A titration period is required to reach optimal dose. Doses of greater than 12 sprays per day are not recommended.

Epidiolex (an oral oil) contains pure cannabidiol (CBD) as the active ingredient. It has been developed to treat some forms of epilepsy, including Dravet syndrome and Lennox–Gastaut syndrome, in children and adults. Epidiolex has been approved by the FDA for use in the United States and has received approval from other regulatory authorities in various countries (32–34). The recommended starting dosage is 2.5 mg/kg by mouth twice daily (5 mg/kg/day).

Marinol (dronabinol) is a synthetic analogue of delta-9-tetrahydrocannabinol (THC). Marinol is used to reduce nausea and vomiting caused by chemotherapy in patients with cancer, as well as to stimulate appetite in patients with HIV/AIDS who suffer from cachexia (decreased body weight and appetite). It is available as oral capsules (32, 35, 36). To treat anorexia associated with weight loss in adult patients with AIDS, the recommended starting dosage is 2.5 mg taken orally twice daily. For nausea and vomiting associated with chemotherapy in adult patients who have not responded to conventional antiemetics, the suggested starting dosage is 5 mg per square meter of body surface area.

Cesamet (nabilone) is another synthetic THC drug for the treatment of nausea and vomiting resulting from chemotherapy in patients with cancer who have not responded adequately to classical antiemetic therapy (32, 33, 37). Each Cesamet capsule contains 1 mg (2.7 μmol) nabilone. The usual adult dosage is 1 or 2 mg 2 times a day.

However, in addition to registered medicinal products for medical purposes, unprocessed cannabis, standardized cannabis preparations, and cannabis preparations made according to the main and official formulas in the pharmacy can also be used. Access to them varies depending on the legislation of a particular state. It is important to note that in order to minimize risks associated with the medical use of cannabis, strict adherence to quality and safety standards is essential. For example, cannabis sold in Germany must abide by the Good Agricultural and Collection Practices (GACP) and Good Manufacturing Practice (GMP) quality standards. Other quality requirements for flower products include compliance with the German Pharmacopoeia (DAB) Monograph, which, among other things, mandates for flower products a deviation of no more than 10% from the declared THC and CBD value; and European Pharmacopoeia requirements. This includes testing results below certain levels of microbiological quality and heavy metals. Compliance with European Pharmacopoeia standards is also essential, ensuring that microbiological quality and heavy metal levels are within safe limits (38). Discrepancies in the concentrations of active ingredients and high doses of cannabis are associated with impairments in cognitive and motor functions, dependence, mental health issues, as well as effects on the lungs and cardiovascular system. Furthermore, cannabis use can cause significant harm to others and lead to increased healthcare costs (39).

Despite the potential benefits of cannabis, undesirable effects have so far limited its medical use. The undesirable effects of cannabinoids and their derivatives can be both short-term and long-term. Short-term effects of cannabinoids and their derivatives may include altered perception, euphoria, increased sense of appetite, drowsiness, decreased blood pressure, dry mouth, and possible side effects such as anxiety, panic, paranoia, and slowed reactions. While long-term use of cannabinoids and their derivatives may be associated with the development of addiction, cognitive and memory decline, poorer mental health, including possible mood disturbances and depression, and an increased risk of developing serious diseases such as cancer and cardiovascular disorders (40–45). Insufficient regulation of medical cannabinoid use programs may contribute to the off-target consumption of cannabis in the population. This could lead to the spread of unregulated recreational use of hemp, potentially negatively impacting health care.

The opinions of researchers on the prospects for use of cannabis for medical purposes vary (46–52). There is a need for further studies with more patients and an evaluation of the long-term effects.

Although cannabis may have positive effects on patients with certain medical conditions, there has been an increase in cases of disorders associated with its use. Excessive cannabinoid vomiting syndrome, for example, is becoming more common (53). Therefore, it is important not only to study the therapeutic potential of cannabis but also to analyze its negative adverse reactions.

Social media has become an integral part of our daily lives and has an impact on various areas, including healthcare. They are an important channel for the dissemination of health information (54). Thus, social networks play an important role in pharmacovigilance and medicine, providing education, support, monitoring, and dissemination of information about health and medical services.

One popular social media platform is Instagram. Compared to other social networks, where there are various features such as chats, statuses, and posting photos, Instagram focuses on photos, videos and posters. This clear direction allows attracting users who are passionate about photography, videos, and posters, making it a unique platform in this area. Instagram provides users with the ability to share photos and videos and communicate with friends. Instagram is also heavily used for commercial purposes, healthcare, and medicine. Hashtags on Instagram help users quickly find content on a specific topic or keyword. Support in the form of “likes” reflects the level of social support or interest in content. However, popularity on social media does not always guarantee a high level of medical competence.

In this regard, pharmacoepidemiological studies have long been a source of data and evidence supporting the safety assessment of approved drugs after they enter the market. The ICH M14 guidelines note that studies including data extracted from websites, blogs, social media, and chats may be insufficient, but they can provide supplementary data for hypothesis generation and contextualizing results (55).

The aim of this study was to investigate the reported efficiency and safety of medical use of cannabinoids in patients using posts on the social media Instagram and analyze the observed trends.

## Materials and methods

### Data source and curation

A qualitative content analysis of cannabinoid posts on Instagram was conducted.

Instagram combines visual content with text, allowing users to share opinions and clear examples of cannabinoid use. In contrast, for example, the X platform focuses on text content, where users post short messages (tweets) up to 280 characters long. It should be noted that Instagram has a significant youth audience, the most vulnerable to cannabinoids, which makes it an important source of data for our research.

Social media listening platform Apify was used to collect data with hashtags as of June 4, 2024 (56). There are several reasons for selecting this platform. Apify integrates with Instagram, ensuring access to data. It also offers flexible data collection settings, enabling the selection of specific hashtags for a more focused analysis. Apify organizes data chronologically, facilitating the examination of current discussions and user opinions at the time of collection. Additionally, Apify can handle big data volumes, essential for analyzing Instagram, where the number of posts and interactions is substantial. The platform supports data export in various formats (Excel, CSV, JSON, etc.), simplifying further processing in analytical tools like RStudio. Apify’s analytical features facilitate the identification of key topics and trends. Importantly, Apify collects only public data, excluding personal information from private profiles. In addition, in recent years, the Web of Science and Scopus databases have presented articles using the Apify platform to analyze data from Instagram and other social networks, which confirms its effectiveness and relevance in scientific research (57, 58).

The posts were extracted using the Apify platform as of June 4, 2024, allowing us to focus on the most relevant information and discussions as of that date. Apify collects posts in chronological order, including those from 2023 and 2024, with some data extending into later periods, in compliance with a systematic approach to data collection.

Instagram hashtags make it easier to find information, expand the reach of content, and allow users to find relevant events and trends, participate in the community, and access a variety of information. For our study, in order to minimize information noise and obtain data only on drugs approved for medical use, we focused on hashtags related to active pharmaceutical ingredients, their derivatives, and registered medicines presented in the British National Formulary or approved by the FDA. We chose the hashtag #Hemp in reference to the active pharmaceutical ingredients derived from hemp and also due to significant user interest (6,308,025 posts). Primary screening revealed that the hashtags #Cannabis, #Marijuana, and #Cesamet were not identified on Instagram as of June 2024, which is related to the platform’s politics and censorship. Instagram has strict rules and algorithms for filtering content that can be linked to drugs or medicines. Under Instagram’s policy, hashtags can be hidden or restricted to prevent the spread of information that goes against community guidelines. This is aimed at complying with regulatory requirements and ensuring the safety of platform users. The study included cannabinoid-related data, including #Cannabinoid, #Tetrahydrocannabinol, #Cannabidiol, #Delta8, #Hemp, #Sativex, #Epidiolex, #Marinol, and #Nabilone.

We selected the first 200 posts for each hashtag for analysis for the following reasons. First, selecting 200 posts is a widely accepted practice in social media research for qualitative analysis (59–63), allowing to focus on popular topics and trends of interest to users. Moreover, the assumption is that most users rarely view content beyond the first 200 images in a normal search (57). Secondly, the hashtag #Hemp had significant user interest, with 6,308,025 posts. In contrast, other hashtags related to registered active pharmaceutical ingredients had fewer posts. Selecting 200 posts allowed for an optimal, in-depth qualitative analysis of the most relevant topics.

In our study, we used the Apify platform, which complies with GDPR requirements. In respect of legality, fairness, and transparency, Apify does not hack or unauthorized access to data but provides tools for legally extracting information from open sources. All data is presented anonymously, without mentioning specific accounts or personal data. Concerning goal limitation, the goal of our study is to evaluate the efficacy and safety of the medical use of cannabis. This is important for the development of medical practice and informing the public about the possible risks and benefits. We collect data, such as the text of posts and hashtags, strictly in accordance with this goal, without the need to process personal data. In relation to data minimization, the Apify platform allows us to collect only the data that is needed to meet research objectives. Concerning accuracy, during the analysis process, we regularly check the data for correctness. With consideration of storage limitation, data collected will only be retained for the time required to achieve the study objectives. Envisaging integrity confidentiality, and security, all data is submitted anonymously. Finally, in respect of accountability, no personal data was used during the work.

By default, Apify collects all available data that it can get from Instagram. Some hashtags were less popular and received fewer than 200 posts. The total number of posts collected by the studied hashtags was 1,592. Some of the collected posts were published a decade ago, which may also explain the diversity in the number of posts across the hashtags under investigation. The data obtained are shown in Table 1. Exclusion criteria was that if multiple posts conveyed the same message or information, only one of them was retained in the analysis. Since a post can contain several of the examined hashtags, after removing duplicates, the number of posts for analysis turned out to be 1,466.

**Table 1:** Distribution of posts with hashtags related to the use of cannabis.

Hashtags	Absolute number of posts included in the analysis (%)	Number of posts identified as of June 2024 (%)
#Cannabidiol	200 (12.56)	611,194 (8.34)
#Cannabinoid	200 (12.56)	225,981 (3.09)
#Delta8	196 (12.31)	174,931 (2.39)
#Epidiolex	197 (12.38)	1,841 (0.03)
#Hemp	200 (12.56)	6,308,025 (86.11)
#Marinol	200 (12.56)	914 (0.01)
#Nabilone	125 (7.86)	183 (0.002)
#Sativex	200 (12.56)	1,646 (0.025)
#Tetrahydrocannabinol	74 (4.65)	194 (0.003)

Posts were analyzed by five attributes: post context type, post content, post visual type, post language, and post sharing type (58). The post context type included the following categories: educational, personal experience, advertising/commercialization, and others (posts that do not fall into any of the three previous categories). The post content was also classified as correct or irrelevant. Information related to cannabinoids was considered correct. Information not consistent with cannabinoids, despite hashtags, was assessed as irrelevant. The post visual type was classified as an image or video (personal photo or video, video, poster). All analyzed posts were divided into five main groups according to the language of the text of the first commentary and general visual elements: English, German, French, Spanish, Japanese, and other languages. The type of post sharing was also considered from the point of view of the author of the post: an individual or organization. The number of “likes” associated with each image at the time of its analysis was also collected.

This study does not apply to specific Instagram users. All data is presented anonymously without mentioning specific accounts.

### RStudio analysis

The analysis of the data obtained from the research was conducted using the RStudio platform (64). Several built-in packages were used for the analysis, including “tm,” “stringr,” “officer,” “tidytext,” and “dplyr.” Initially, the data were processed to improve their quality and relevance for analysis. This included removing punctuation, numbers, and emojis, achieved using the ‘gsub’ function in R. Although common in social media posts, these elements do not contribute to meaningful text analysis and may distort the results. Further refinement involved excluding common words-specifically personal pronouns, adjectives, and conjunctions-using the ‘remove_words’ function from the “tm” package. This step is critical as it eliminates noise from the data, allowing for a clearer focus on significant terms that reflect user sentiment and thematic content related to cannabinoids. To address variations in word usage, we merged repeated words with different capitalization. This normalization process was facilitated by the “tidytext” package, ensuring that variations of the same word were counted as a single entity, enhancing the accuracy of frequency counting. Words containing ‘hashtags (#)’ were filtered out using regular expressions (‘regex’) from the “stringr” package. This ensured an accurate word frequency count, making sure only relevant terms were included in the analysis. We calculated the frequency of each word present in the cleaned dataset by creating a term-document matrix, which allowed us to quantitatively assess the occurrences of each word. As a result, the “Top 50 most frequently used words” and “Top 50 most frequently co-occurring hashtags” were identified.

## Results

A comprehensive review of the content of posts by studied hashtags was carried out, analyzing the distribution by type of context, correctness of information, visual type, language and authorship. The results are shown in Table 2.

**Table 2:** The main content trends of posts containing hashtags (#Cannabinoid, #Tetrahydrocannabinol, #Cannabidiol, #Delta8, #Hemp, #Sativex, #Epidiolex, #Marinol, and #Nabilone) in the use of cannabis and cannabinoids for medical and recreational purposes on Instagram.

Category	Absolute number of posts, %	% of relevant posts
The post context		
Educational	293 (19.99)	100.00
Personal experience	375 (25.58)	96.00
Advertising/commercialization	485 (33.08)	91.75
Others	313 (21.35)	31.63
The post content		
Correct	1,199 (81.79)	
Irrelevant	267 (18.21)	
The post visual type		
Personal photo	330 (22.51)	66.36
Personal video	72 (4.91)	81.94
Poster	880 (60.03)	81.59
Video	184 (12.55)	85.87
Language		
English	1,037 (70.74)	78.98
German	202 (13.78)	92.08
French	50 (3.41)	90.00
Spanish	49 (3.34)	87.76
Japanese	17 (1.16)	70.59
Others	111 (7.57)	81.98
The author of the post		
An individual	533 (36.35)	73.92
Organization	933 (63.65)	86.28

Analysis of overall content relevance revealed that the majority of Instagram posts (81.79%) related to cannabis and cannabinoid hashtags are relevant. This indicates a high level of interest and attention to cannabis topics among users. The presence of irrelevant content (18.21%) is also important to consider, as it shows the presence of some volume of posts that do not fully correspond to the main topic, which may be caused by the widespread use of hashtags to attract attention or the inclusion of related topics.

Based on the analysis of content on Instagram, there are several main trends in posts related to the use of cannabis and cannabinoids. Educational posts occupy almost 20% of the total number of publications and are highly relevant. These materials highlight the importance of information related to research and clinical trials, as well as the health effects of cannabinoids.

The main topics presented in the educational posts cover a wide range of aspects. First, various cannabinoid drugs such as Sativex, Epidiolex, Nabilon, and Marinol are discussed, including their effects and side effects. A comparison of these drugs demonstrates the variety of their uses in the treatment of various conditions, for example, chronic pain, epilepsy, and symptoms of multiple sclerosis.

Second, attention is focused on the potential therapeutic properties of cannabinoids such as cannabidiol, which is being actively investigated for the treatment of chronic pain, fibromyalgia, and anxiety disorders. Cannabidiol has also been shown to be effective in improving sleep quality and reducing insomnia, and its anti-inflammatory properties may aid in recovery from exercise.

Third, the posts highlight the importance of information from medical professionals, including advice and guidance on the use of cannabinoids to optimize treatment and management of symptoms of various diseases.

In addition, educational posts feature conference results, article publications, and reports, suggesting a growing interest in cannabinoids and their role in modern medicine. New approaches to cannabinoid use are discussed. The possibility of their use in combination therapy with other treatment methods is noted. All of these topics highlight the need for further research to confirm their efficacy and safety.

Posts containing information about personal experience make up more than a quarter of all publications and have a high level of relevance (96%). This points out to the greater popularity of the personal stories of users sharing their experiences with cannabis for medical purposes, describing their results and effects. In some cases, posts are presented in the form of personal photos or videos that describe their condition after using cannabinoids.

Posts of an advertising and commercial nature occupy the largest share (33.08%) among all categories, as well as a high level of relevance (91.75%). This highlights the strong promotion and commercial use of cannabis, including advertising of various products and brands. Along with information on the medical use of cannabis, posts promoting various forms of recreational cannabis, such as chewing gum, food, drinks, vapes, etc., are widely presented.

Other posts account for 21.35% of the total number of publications, but have a significantly lower percentage of relevance (31.63%). They include diverse content that does not fall into other categories, perhaps due to a less clear association with the topic of cannabis and cannabinoids. This category included posts on the legalization of cannabis, information on laws and regulations regarding the use of cannabis in various countries and regions, accessibility, and financing. Videos and photos of people using cannabis for recreational purposes were also allocated to this category.

Consequently, educational posts have high relevance and constitute a significant portion of content (19.99%), reflecting the importance of the educational aspect in cannabis discussion. The personal experience of users is also popular and highly relevant (96%), indicating a large number of stories and reviews from real people. Advertising and commercial posts dominate the total (33.08%) and have a high relevance (91.75%), highlighting the commercial promotion of cannabis. It is worth noting that the legal status of recreational cannabis, including chewing, food, drinks, vapes, etc., depends on the country and even the region within the country. Laws can vary significantly from country to country. Other posts have a low level of relevance (31.63%), which may indicate a variety of content that is not always closely related to the main topic. These findings provide insight into how different aspects of cannabis use are represented and discussed on the social network Instagram.

The authors also analyzed different types of visual content on Instagram related to cannabis. Posters dominate all types of content, accounting for more than half of all posts (60.03%). The percentage of relevant posts among them is also high and amounts to 81.59%. Videos make up 12.55% of the total number of posts and also represent a high percentage of relevance (85.87%). Personal videos and personal photos have a high proportion among all posts and also show a high level of relevance, especially personal videos (81.94%).

The authors analyzed the language of the Instagram posts they were posted on. Posts in English account for 70.74% of the total number of posts and dominate the category under study. The percentage of relevant posts among them turned out to be 78.18%. German is the second most common language (13.78%). Posts in German have a high percentage of relevance—92.08%. French and Spanish occupy a significant place among the languages that are highly relevant. Japanese posts account for 1.16% of the total number of posts, with a sufficient percentage of relevance among languages (70.59%). The category “others” includes posts in languages such as Arabic, Chinese, Croatian, Danish, Dutch, Farsi, Finnish, Georgian, Greek, Indonesian, Italian, Korean, Macedonian, Polish, Portuguese, Romanian, Slovenian, Suomalainen, Swedish, Thai, Turkish, and Ukrainian. A good level of relevance was identified for these posts, which emphasizes the importance of a multilingual approach in content analysis.

Analysis of data by authors showed that the majority of posts in the studied category are published by organizations (63.65%). These posts also show a higher percentage of relevance (86.28%) compared to posts posted by individual users (73.92%). It revealed that organizations are taking a more targeted and professional approach to creating cannabis-related content, making their posts more informative and relevant to audiences. These accounts play a key role in informing and promoting cannabis content on Instagram.

Table 3 shows the posts with the most “likes.” Posts with personal stories in English, especially Turkish (high emotional engagement), received a significant number of “likes” which indicates high audience engagement. Educational posts in English and Spanish are also popular, pointing out the importance of providing scientific information on research and practical aspects of cannabis use. Advertising content (German, Portugal) also attracts the attention of the audience. In the “other” category, posts about news related to the legalization of medical marijuana in South Korea received a high “like” rating.

**Table 3:** Posts with the most “likes.”

N	The post context	Number of “likes”
1.	A personal post (in Turkish) from the mother of a child with refractory epilepsy describing the ongoing battle with the disease.	1,072
2.	A post dedicated to personal experience (in Turkish), where a mother of a child with refractory epilepsy shares that for the next 3 months they will be receiving Epidiolex for treatment.	682
3.	An education-related post (in Spanish) reports that the research team obtained new data on the therapeutic potential of cannabinoids in epilepsy.	642
4.	A post from the “other” category (in Polish) reports that South Korea has legalized medical marijuana, allowing doctors to prescribe controlled, “non-hallucinogenic” doses of it.	584
5.	An education-related post (in Spanish) mentioning that a team of researchers discovered a novel mechanism of action for cannabidiol.	539
6.	A German advertising post reports that while the new Cannabis Act does not yet allow cannabis sales in specialized stores, major beverage manufacturers are already planning to launch cannabis products for retail.	474
7.	An education-related post (in English) reports that according to research published in Frontiers in Cardiovascular Medicine, patients with a history of stroke are not at an increased risk of cardiovascular complications after using nabiximols (Sativex).	451
8.	An education-related post (in English) affirming that patients’ CBD consumption leads to pain reduction.	406
9.	A post related to a personal experience (in Turkish) in which the mother of a child with refractory epilepsy shares the results of successful treatment.	395
10.	An education-related post (in English) highlights the increasing recognition of CBD as a promising treatment in epilepsy research.	371
11.	An education-related post (in English) reports that a new clinical trial has found CBD to significantly alleviate symptoms in patients with gastroparesis.	336
12.	An education-related post (in Spanish) reports that a research team has uncovered the psychoactive mechanisms in cannabis.	316
13.	The post belonging to the category “other” (in English) informs that a House committee recently passed an amendment to ban any level of THC in ingestible hemp products.	311
14.	An education-related post (in English) reveals that about 80 percent of multiple sclerosis patients with severe spasticity experience significant symptom relief after using the cannabis spray Sativex.	306
15.	A personal experience post (in English) describes that a mother found her son had a seizure on the floor after his dose of Epidiolex was reduced; she was concerned about the treatment status.	290
16.	An education-related post (in English) highlights that current research shows significant variability in CBD product quality and dosing, with the composition of many supplements not matching their labels.	269
17.	A Portuguese advertising post emphasizes that negativity against marijuana comes from a lack of information and claims that marijuana can save lives.	267
18.	An education-related post (in Polish) discusses the high popularity of cannabidiol (CBD) and critiques the often misleading marketing.	265
19.	In a personal post (in English) a cancer patient talks about the ongoing struggle with the side effects of chemotherapy, taking Marinol and the high costs of treatment, thanks the nurses for their support and hope to remain strong for the upcoming fundraising for the drug.	251
20.	A personal post (in English) describes how THC, prescribed for chronic pain and mental health disorders, has significantly aided in the recovery process and mental wellbeing.	248

**Figure 1:**
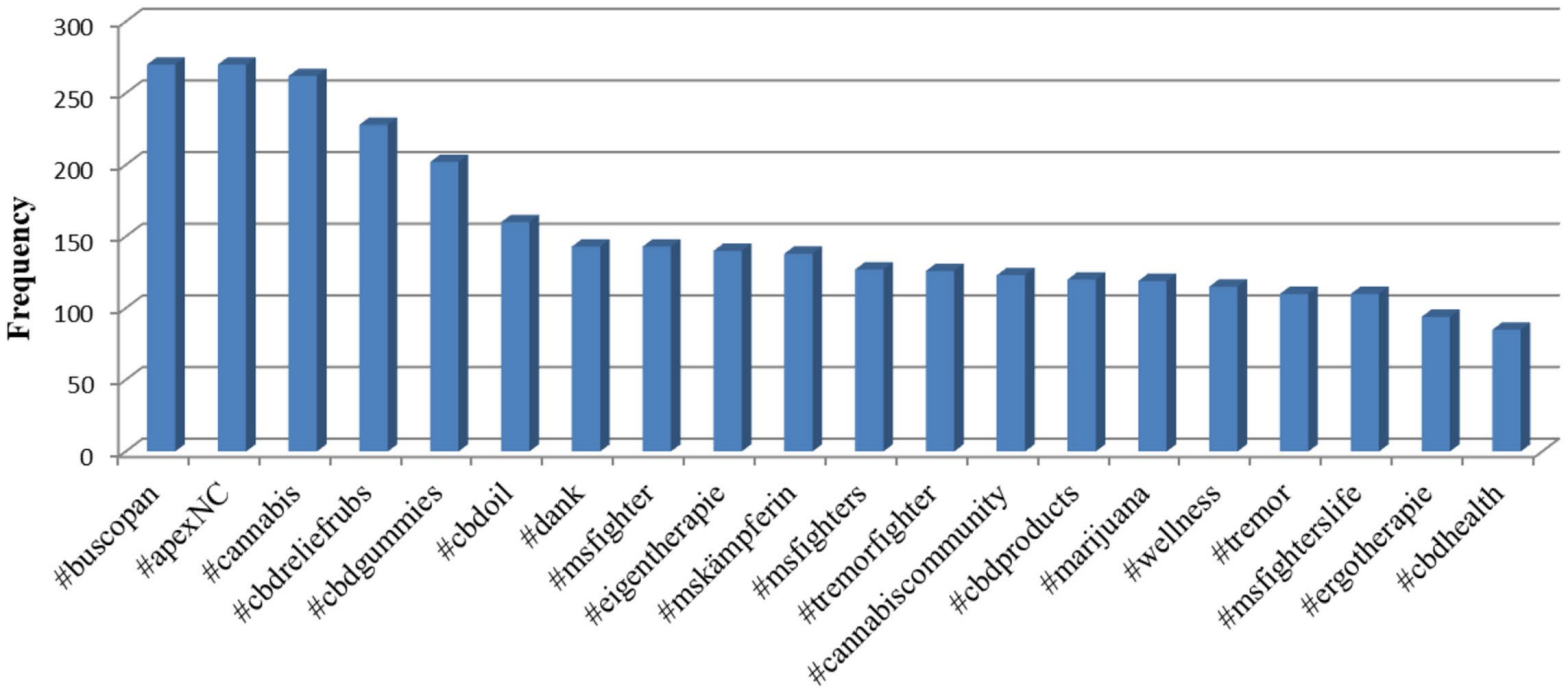
Co-occurring hashtags.

The co-occurring hashtags are shown in Figure 1. The hashtags provided show that the focus is on cannabis-based products, especially CBD, and related topics. Hashtags such as #cannabis, #cbdreliefrubs, #cbdoil, and #cbdgummies highlight interest in different forms of CBD and its application for health relief. In addition, hashtags such as #msfighter and #mskämpferin point to a link between cannabis use and the fight against multiple sclerosis.

**Figure 2:**
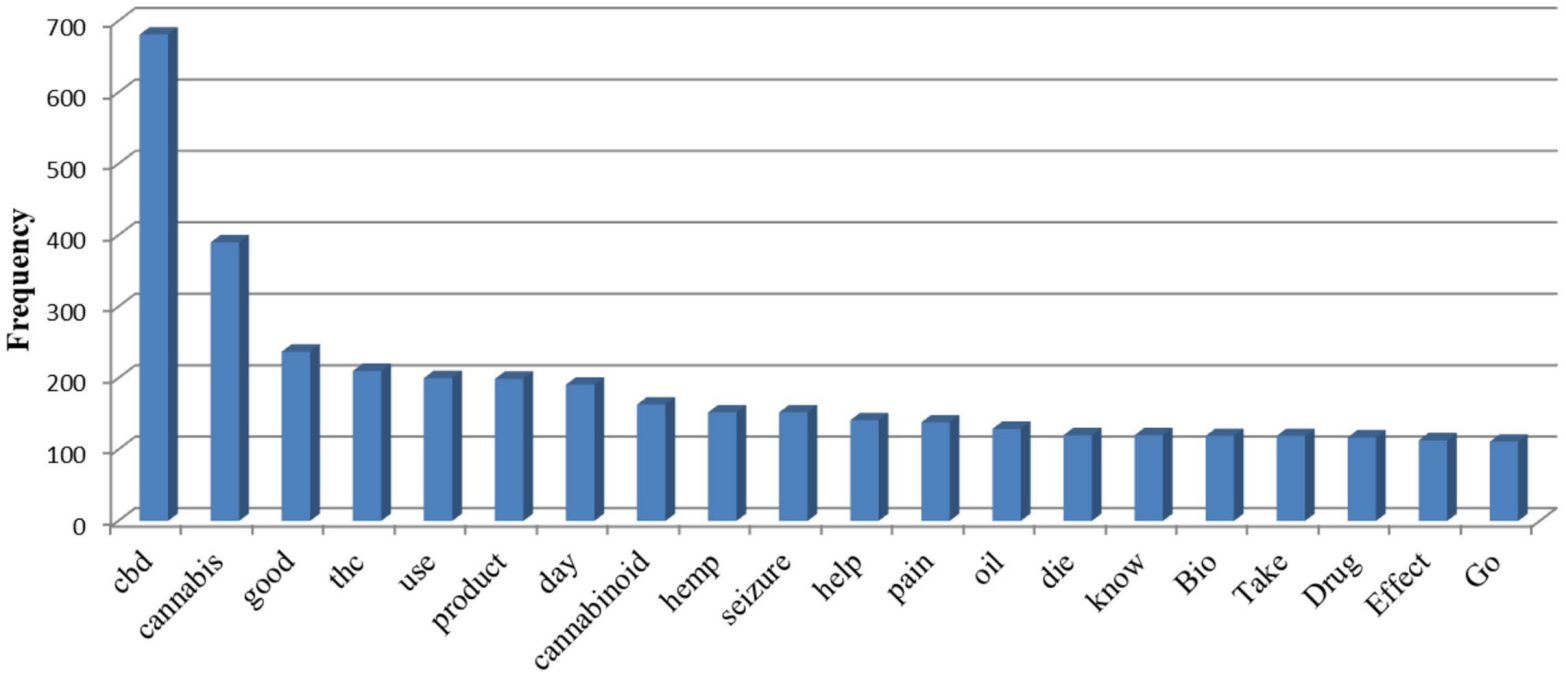
Most common words in posts.

The most common words in the posts are given in Figure 2. There is a preponderance of keywords related to CBD and cannabis, THC (tetrahydrocannabinol), in the data presented. It is indicating significant interest in these substances, their uses, and products. It can also be observed that words such as “good,” “help,” and “pain” are associated with positive effects and medical use, which may indicate the popularity of discussions about how CBD and cannabis help in relieving symptoms and improving the overall health of patients.

As a result of the analysis, the most frequently used co-occurring hashtags, with 50 or more mentions, were identified. They were divided into three main thematic groups: “cannabis medicines and substances,” “health and wellness,” and “community and lifestyle.” The resulting data are presented in Table 4.

**Table 4:** Most frequently used co-occurring hashtags.

Groups of co-occurring hashtags	Frequency of occurrence (mean and standard deviation)
Cannabis medicines and substances	153.10 ± 77.49
Health and wellness	103.16 ± 42.67
Community and lifestyle	89.76 ± 44.60

The group “cannabis medicines and substances” includes hashtags related to specific cannabis products, their medical properties, and their uses. This group focuses on medicinal products containing cannabinoids as well as those available on the market. Using such hashtags enables Instagram users and others to find information about cannabis and related products. The second group of “health and wellness” hashtags focuses on the topics of health, wellbeing, and the use of cannabinoids to treat various diseases and maintain overall health. These hashtags reflect interest in the medical and therapeutic aspects of cannabis use, especially in the context of the management of diseases such as epilepsy and multiple sclerosis. They help users find communities and resources that support their health and wellbeing. The third group of hashtags related to “community and lifestyle” creates a space for sharing experiences, self-expression, and mutual support. These hashtags are important for people facing chronic diseases or looking for ways to improve their quality of life. They promote supportive networking and awareness.

**Figure 3:**
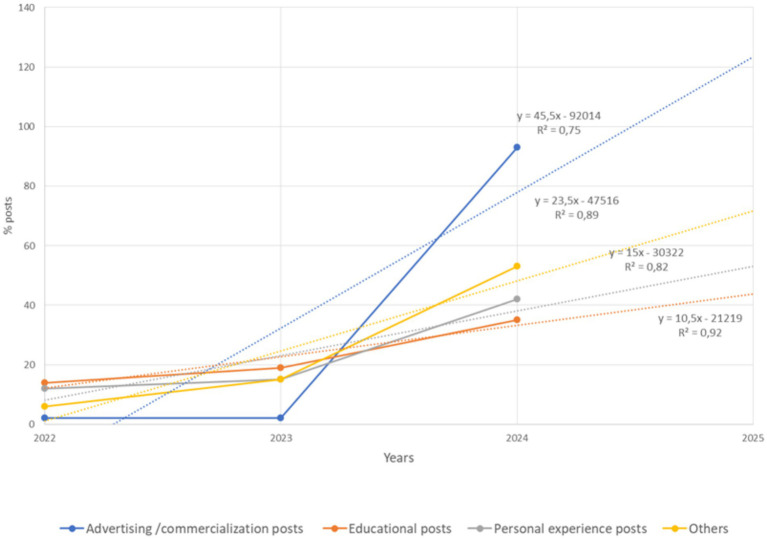
Dynamics of the number of posts about cannabinoids on Instagram by category (2022–2024).

Dynamics of the number of posts about cannabinoids on Instagram by category (2022–2024) are presented in Figure 3. The largest increases in advertising/commercialization posts are found, while education and personal experience posts show steady growth, with the most significant jump in personal experience posts. The high number of advertising posts indicates a significant promotion of cannabinoids in the market. This may be due to increasing commercialization and increased interest in the product. The number of educational posts is increasing, reflecting efforts to inform the audience. The medical community is gradually increasing the volume of scientifically based content, but the pace of this growth remains moderate. The increased number of posts with personal stories may indicate an increase in interest in cannabinoids. People are more likely to share their experiences, contributing to the normalization of the topic and strengthening public perception. Category “other” shows consistent growth, indicating an expansion of the discussed cannabinoid aspects. This may indicate a deeper study and a diversity of views on the topic.

Thus, the medical community should focus on increasing the number of educational posts to improve health literacy. With the rise of advertising and personal experience posts, there is a risk that audiences may be influenced by unverified information or advertising content. To counteract this, the medical community must actively create more educational content that provides accurate, evidence-based information about cannabinoids. These posts can eliminate common misconceptions, provide information about safe use, educate users about potential risks and benefits, and help form an informed attitude toward their use.

The identified main topics of discussion, according to the analysis, are presented below.

## Discussion

Educational and personal posts indicate that Sativex, Epidiolex, Marinol, and Nabilone are used in the treatment of various diseases that are difficult to treat with other medications or are not treatable at all. These diseases include epilepsy with Lennox–Gastaut and Dravet syndromes, multiple sclerosis, cancer and HIV cachexia, nausea and vomiting caused by chemotherapy, chronic pain syndrome, and dementia. The presented review offers a ray of hope for effective treatment, but the manifestation of their side effects is concerning. Another issue is that clinical studies are ongoing, and only their completion will allow for definitive conclusions regarding the safety, benefits, and risks of these drugs. However, preliminary studies show that educational and personal posts largely align with clinical data.

We present an analysis of educational and personal posts and clinical studies that demonstrate both the efficacy and potential side effects of these compounds. Additionally, the analysis addresses concerns regarding advertising and commercial posts on Instagram related to the advocacy of recreational cannabis.

### Educational posts and personal experience

#### Epilepsy with Lennox–Gastaut and Dravet syndromes

Epilepsy is a common neurological disorder, the treatment of which is often difficult due to resistance to therapy. Severe forms of epilepsy include Dravet syndrome and Lennox–Gastaut syndrome. Dravet syndrome is a form of epileptic encephalopathy, manifested in the first year of life by treatment-resistant seizures (febrile and afebrile), mental retardation, and myoclonic paroxysms. Lennox–Gastaut syndrome is a serious epileptic encephalopathy in which epileptiform changes can contribute to cognitive impairment (65).

Educational and personal experience-based posts describe the efficacy and side effects of using cannabinoids, cannabidiol, and Epidiolex to treat seizures in patients aged 2 years and older suffering from two rare and severe forms of epilepsy with Lennox–Gastaut and Dravet syndromes. The information presented in these posts correlates with data from recent scientific publications in clinical practice.

For example, meta-analysis data from six randomized clinical trials supports the efficacy of oral cannabidiol as a self-therapy and complementary therapy for seizure control in Dravet syndrome and Lennox–Gastaut syndrome (66). A review of eight randomized controlled trials found that cannabidiol may reduce attack rates in Dravet syndrome and improve overall treatment experience, but also causes decreased appetite and may be associated with adverse mental events (67). An analysis of 50 studies showed stable efficacy and an acceptable safety profile of cannabidiol in short-term use for the treatment of drug-resistant epilepsy; however, efficacy may decrease and the incidence of side effects increase with long-term use (68).

Scientific publications indicate that сannabidiol use is associated with increased incidence and severity of adverse events, including serious side effects and cases leading to dose withdrawal or reduction. Further deeper studies are needed to determine the optimal and safe dosage of cannabidiol in the treatment of epilepsy (69). Cannabidiol used as adjunctive treatment for Dravet syndrome has shown efficacy in reducing the incidence of seizures compared to placebo but is also associated with an increased risk of adverse events such as drowsiness and decreased appetite (70).

Some posts also noted the presence of autistic disorders along with epilepsy. There is evidence that cannabis products can reduce symptoms of autism spectrum disorder, such as hyperactivity, bouts of aggression, sleep problems, and anxiety, as well as improve cognitive function and social interaction. Further studies of these effects and side effects are needed (71).

There is extensive information that Δ9-THK acts as a partial agonist for the CB1 and CB2 receptors belonging to the endocannabinoid system. In addition to this, it can bind to other molecules affecting nervous excitation and inflammatory processes in the nervous system. These effects provide its potential in the therapy of neurological conditions such as epilepsy (72).

Thus, Instagram posts describing the use of cannabinoids to treat epilepsy reflect scientific evidence of the efficacy of cannabidiol in treating seizures in Dravet and Lennox–Gastaut syndromes. However, despite the positive results, adverse effects and the need for further research to optimize therapeutic recommendations and dosages should be considered.

#### Multiple sclerosis

Multiple sclerosis is a chronic autoimmune disease of the central nervous system that affects people predominantly in their 20s and 40s and is one of the leading causes of non-traumatic disability among adults. Timely diagnosis and effective therapy of the disease can significantly reduce the number of patients with disabilities. The incidence of multiple sclerosis varies and, on average, reaches 8–10 new cases per 100,000 people, with more than 700,000 people living with this diagnosis in Europe and more than 2.5 million worldwide. Despite active research, there are no drugs that completely cure multiple sclerosis. Available treatment focuses on slowing disease progression, reducing exacerbation rates, relieving symptoms, and reducing disability (73).

Spasticity in diffuse sclerosis often causes significant difficulties with movement and can be effectively facilitated by Sativex (nabiximols), which contributes to a decrease in muscle tone and improved coordination (32). This is mentioned in educational posts and descriptions of personal experiences on Instagram. Patients share on social media their positive experience of Sativex use, noting a significant reduction in spasticity and improved overall coordination.

Data from clinical studies confirm the effectiveness of sativex in reducing spasticity and improving the quality of life of patients with scattered sclerosis. For example, an analysis by Dykukha et al. showed that nabiximol spray has no detrimental effect on cognition in patients for up to 12 months of observation (74). Research by Markovà et al. demonstrated significant clinical improvement in spasticity with sativex as adjunctive therapy (75).

In addition, Haupts et al. noted that Sativex significantly improves spasticity symptoms such as pain and bladder problems (76). D’hooghe et al. reported that more than 60% of patients who started Sativex treatment after failures with other remedies noted a significant improvement in spasticity symptoms (77).

Also, Patti et al. and Nicholas et al. confirmed the efficacy of Sativex in reducing spasticity and improving associated symptoms in patients, even in those who did not respond to conventional antispastic drugs (78, 79). It is worth noting that in a number of cases, side effects and difficulties with the availability of the drug can lead to its cancelation or violation of the treatment.

It is reported that patients with multiple sclerosis show changes in the expression of CB1 and CB2 receptors, which may explain the therapeutic potential of cannabinoids in this pathology (80).

Sativex is thus presenting an effective and safe means for alleviating the symptoms of spasticity in patients with disseminated sclerosis, improving their quality of life, and facilitating movement. These findings are confirmed by both scientific research and personal experience of users, highlighted in posts on Instagram. At the same time, the side effects of the drug are usually mild and insignificant, which also emphasizes its safety in long-term therapy.

#### Cancer and HIV-cachexia. Nausea, vomiting caused by chemotherapy

Cancer cachexia is a complex syndrome characterized by permanent loss of muscle mass and a negative balance of protein and energy, which cannot be compensated by normal nutrition and is caused by a combination of reduced appetite and impaired metabolism. Cachexia syndrome can accompany the course of many diseases, including AIDS. However, the mechanisms leading to the onset of this syndrome may have features depending on the underlying disease (81).

It should also be noted that modern chemotherapy has significantly increased the survival rate of patients with cancer. However, such effective treatments are often accompanied by side effects and complications that make them difficult to fully implement and reduce the quality of life of patients (82). The most common side effects of chemotherapy are nausea and vomiting. Highly emetogenic therapy often leads to dehydration, anorexia, electrolyte disorders, and damage to the mucous membrane of the esophagus and stomach (83).

There is evidence that the endocannabinoid system regulates nausea. This supports the use of cannabinoids in the treatment of nausea and vomiting (84). Additionally, experimental data exist regarding the appetite-stimulating properties of cannabis (85).

Instagram users in the studied posts write about the symptoms of cancer and HIV-associated cachexia, nausea, and vomiting during chemotherapy. In the context of the treatment of cachexia, the use of dronabinol (marinol) is mentioned, which is used to improve appetite and maintain body weight in patients suffering from this condition. There are posts related to the effectiveness of dronabinol and nabilone for the treatment of nausea and vomiting caused by chemotherapy.

While evidence from clinical studies suggests that cannabinoids, including dronabinol and nabilone, can effectively increase appetite in patients with cachexia and cancer and reduce nausea and vomiting with chemotherapy. However, their impact on overall quality of life remains limited, and side effects such as drowsiness, dizziness, and dry mouth can be significant. Moreover, research evidence suggests that while cannabinoids may help manage appetite, their effectiveness in treating depression, anxiety, or stress needs further investigation (86–89). More research is needed to assess the long-term effects and benefits of these drugs.

The therapeutic potential of cannabinoids has attracted considerable attention in the field of oncology. As research progresses, various studies reveal the mechanisms through which cannabinoids may contribute to the treatment of cancer pathologies. Mobaleghol et al. have demonstrated that a nanoemulsion containing cannabis extracts has potential effects for the treatment of glioblastoma in both *in vitro* and *in vivo* studies (90). Specific molecular targets of phytocannabinoids in breast cancer have been identified through *in silico* and *in vitro* studies, indicating their potential therapeutic contribution (91). Besser et al. have found that a combination of cannabinoids targets acute lymphoblastic leukemia of NOTCH1 mutation-induced T cells by modulating an integrated stress response pathway (92). A study by Musa et al. showed that a cannabinoid-enriched product inhibits myeloma cell function by regulating telomeres and the TP53 gene (93). An *in silico* analysis by Du Plessis et al. predicts that cannabidiol may act as a potential inhibitor of the MAPK pathway in colorectal cancer, suggesting its therapeutic value (94). However, Wang et al. note that although cannabidiol has been shown to have antitumor activity, its use in combination with effector cell-based immunotherapy may reduce the antitumor efficacy of activated NK cells (95). Wright presented a comprehensive review of direct cannabinoid targets, highlighting the effects on various G protein receptors and cation channels (96). Mashabela et al. showed that the anticancer and antiproliferative effects of cannabidiol can be both receptor-dependent and receptor-independent (97). Suzuki et al. investigated how cannabigerolic acid (CBGA) inhibits the TRPM7 ion channel, suggesting its potential impact on cellular functions in cancer, kidney disease, and stroke (98). Thus, research data show that cannabinoids can target a wide range of molecular targets and biochemical pathways against the background of various forms of cancer.

#### Chronic pain syndrome

Chronic pain syndrome impairs the quality of life of patients, includes two types of pain: nociceptive pain from tissue damage and neuropathic pain associated with damage to the nervous system. This condition can occur in various diseases and injuries, and its treatment requires an integrated approach.

The endocannabinoid system plays a significant role in the perception of pain of various origins. Cannabinoids have demonstrated antinociceptive effects and activity in neuropathic pain (99, 100).

Studies show that cannabinoids help Instagram users to alleviate chronic pain syndrome. There are also posts about the use of cannabinoid drugs in veterinary practice to relieve pain in pets. The scientific literature also presents these effects (101, 102).

However, systematic reviews and meta-analyses suggest that cannabinoids, despite positive user feedback, show limited efficacy in alleviating chronic pain and improving physical functioning and sleep quality. Studies also point to the possibility of temporary side effects such as dizziness and mood changes (103–105). More clinical studies are needed to better assess the benefit and safety of cannabinoids in the treatment of chronic pain syndrome.

#### Dementia

Dementia is accompanied by deterioration in memory, speech, and cognitive functions, and its main causes are Alzheimer’s disease, vascular dementia, and Parkinson’s disease (106). Current treatments have only moderate effects and may have significant side effects (32).

There is evidence that cannabinoids have potential therapeutic efficacy in neurodegenerative diseases (102, 107).

In the investigated Instagram posts, users share their experiences with cannabinoids to manage dementia symptoms. The need for a cautious approach is mentioned, as some patients may experience side effects, such as changes in behavior.

Based on clinical studies, cannabinoids show potential to improve neuropsychiatric symptoms in patients with dementia, but the overall assessment of the quality of these studies remains low. For example, a study of nabilone demonstrated its effectiveness in reducing arousal in patients with Alzheimer’s disease, but the need for careful monitoring of side effects remains urgent task. More research is needed to definitively assess the real-world efficacy and safety of cannabinoids in dementia (108, 109).

The results of recent studies indicate significant therapeutic potential of cannabinoids and their derivatives for the treatment of various neurological diseases, including dementia, ischemic stroke, and Alzheimer’s disease. Basavarajappa et al. consider the potential of little-studied marijuana phytocannabinoids for the treatment of neurological diseases. The authors emphasize that these compounds may exert antioxidant, anti-inflammatory, and immunomodulatory properties (110). Raïch et al. investigate the effect of cannabidiol on CB1 and CB2 receptors in ischemic stroke. The authors found that cannabidiol administration could reduce neuronal damage caused by stroke in experimental animal models (111). Hickey et al. focus on the ability of cannabidiol to modulate oxidative stress and neuroinflammation—key processes in Alzheimer’s disease. According to the authors, the antioxidant and anti-inflammatory properties of cannabidiol may slow down the neurodegenerative processes associated with this disease (112). In a review by Broers et al., the possibilities of using cannabinoids to treat behavioral symptoms of dementia, such as agitation and aggression, are being considered. Low doses of synthetic tetrahydrocannabinol were found to be ineffective; however, more recent studies using high doses of tetrahydrocannabinol/cannabidiol have demonstrated encouraging results, showing efficacy and safety for older patients (113).

Thus, although the investigational compounds require additional study and monitoring of side effects, the promising properties of cannabinoids, such as anti-inflammatory, antioxidant, and neuroprotective effects, open up new possibilities for their clinical application in neurology.

#### Adverse effects of cannabionoids

Cannabinoids are used to treat various diseases, but their use is limited by their narcotic properties. Long-term cannabis use can lead to addiction syndrome. It should be noted that the addictive component is THC, so non-standardized products pose a health risk, as they may exacerbate side effects. In addition, cannabinoids can cause a variety of other adverse effects.

Users share their experiences with various cannabinoids such as Sativex, Epidiolex, Marinol, and Nabilone and their side effects in the Instagram posts investigated. While educational posts help users better understand how these drugs work and what side effects may be.

An extensive analysis of studies on the safety and efficacy of cannabinoid drugs has lately been conducted. Thus, Bajtel et al., in a meta-analysis, showed that Nabilone causes more cases of drowsiness, dizziness, and dry mouth compared to placebo, while dronabinol is associated with an increased incidence of dry mouth, dizziness, and headache (87). This suggests that further studies are needed to better assess their safety profiles.

In studies conducted by Chesney et al., cannabidiol is found to be well tolerated and causes few serious side effects (114). Despite this, research in this direction continues.

Prieto González et al. noted that nabiximol oral mucosal spray has an acceptable safety profile in the treatment of spasticity and chronic pain, as well as a lower rate of discontinuation due to adverse events compared to other types of pain syndromes (115). An analysis by Wieghorst et al. found a slight negative effect of cannabinoid drugs on cognitive function at low and moderate doses of THC, but long-term use can still adversely affect cognitive abilities (116). Huestis et al. reported cannabidiol to be effective in serious conditions such as Dravet and Lennox–Gastaut syndromes. At the same time, possible side effects and interactions with other medications must be considered before using cannabidiol outside the approved indications (117). According to Zhou et al., analysis of the data revealed that the side effects of Epidiolex mainly coincide with those indicated in the instructions; however, new potential effects, such as cluster seizures and overactive pharyngeal reflex, require additional study (118). Georgieva et al. demonstrated that Epidiolex is well tolerated and highly effective in the long-term treatment of refractory epilepsy, despite the side effects that most often occur in the first months of therapy (119). These studies highlight the need for continued research to better assess the safety of cannabinoid drugs.

In conclusion, it should be noted the risks of other side effects of cannabinoids, as presented in the instructions for use of registered drugs, such as salivation, urinary incontinence, lethargy, hypothermia, mydriasis, hyperacusis, increased serum alkaline phosphatase levels, consciousness disturbances including seizures, ataxia, depression, anxiety, vocalization, as well as symptoms such as diarrhea, vomiting, bradycardia, or tachycardia (32–37).

### Advertising and “other”

Advertising and commercial posts on Instagram are widely associated with the promotion of recreational cannabinoids, such as chewing gum, chocolate, granola, drinks, vapes, etc. In the posts, the benefits of recreational products are noted as relieving tension, anxious thoughts, feelings of wellbeing, anxiety, and depression, improving mood, euphoria, solving problems with falling asleep, pain relief, increasing sexuality, etc.

Meanwhile, scientific medical publications provide only limited evidence that cannabinoids are effective in treating anxiety disorders, reducing symptoms of PTSD and short-term sleep disturbances. The development of adverse events against the background of taking cannabidiol in some mental disorders has been reported (48). It should be noted that there is a risk of increased cannabis consumption among adolescents and long-term negative effects on their health.

Saavedra et al. point out that cannabis-based compounds can significantly affect the central nervous system in children and adolescents, causing both temporary mood changes and long-term cognitive and sensory processing impairment (120). Lim et al. note that the prevalence of cannabis smoking among adolescents in the United States and Canada increased from 2013 to 2020, necessitating additional preventive measures and strategies to counter this trend (121). Schmidt et al. found that cannabis use was associated with an increased risk of suicidal behavior and attempts among adolescents, particularly with increased frequency of use (122). Allaf et al. emphasize that the legalization or decriminalization of cannabis leads to a significant increase in cases of acute poisoning, especially among pediatric patients, which emphasizes the need for further monitoring and assessment of the impact of changes in legislation on public health (123).

These studies highlight the need to raise awareness of the possible risks and side effects of cannabinoid products, especially among adolescents, and to implement preventive measures and strategies to reduce the negative effects of their use. Moreover, advertising posts often focus on positive aspects, ignoring potential risks and side effects, which reduce awareness of possible harmful consequences. Moreover, advertising can reinforce stereotypes of cannabis as a harmless entertainment substance, ignoring potential health and social risks.

Bahji et al. found that cannabis withdrawal syndrome is common in those who regularly use cannabis (124). This highlights the need to consider this risk when counseling and supporting patients who reduce cannabis use. A link has been identified between the abuse of synthetic cannabinoids and various cardiovascular diseases, which emphasizes the importance of early detection and effective management of these conditions (125). McCartney et al. showed that delta (9)-THA impairs driving quality and cognitive skills, with impairments likely to persist up to 7 h after consumption (126). Acute cannabis use has been reported to result in mild to moderate cognitive impairment, particularly in verbal learning and working memory (127). Preuss et al. noted that cannabis use increases the risk of traffic accidents, especially at high blood THC concentrations (128). Cannabis use leads to a rapid decrease in driving confidence, which is associated with a decrease in driving ability in a simulator and an increase in reaction time. These effects are seen in both regular and occasional drivers (129). A significant increase in cannabis use and its strength of action has been recorded in Europe from 2010 to 2019, accompanied by a rise in the number of cases of cannabis-related problems being treated (130). This highlights the need for increased monitoring and improved data analysis to better assess the impact of changes in cannabis regulation.

Fischer et al. developed 10 recommendations to reduce the risks associated with cannabis use (131). These recommendations include abstaining from early onset and frequent use, preferring low-THC products, avoiding synthetic cannabinoids and non-smoking use methods, and refraining from driving under the influence of cannabis.

These studies highlight the importance of awareness of the risks associated with cannabis use, particularly among young people, and the need to implement preventive measures to reduce the negative effects.

Our findings are consistent with those of other researchers, such as Walker et al., which noted a significant increase in discussions about Delta-8 THC on Twitter from 2020 to 2022, especially through online retailers (132). They also showed significant overlap with content about cannabidiol and other cannabinoid products. To ensure a balanced discussion and inform the public, it is important that public health researchers actively monitor these trends and promote adequate Delta-8 recommendations on social media platforms.

In the “other” category, Instagram users actively discussed the problems of legalizing medical cannabis drugs, access to them for patients, and affordable prices. Instagram users’ discussions are consistent with literature data highlighting the need to address the legalization and financial inclusion of cannabis-based medicines (132, 133).

Martin et al. emphasize the importance of ensuring access to safe, effective, and affordable cannabis-based medicines for treating diseases that do not respond to traditional methods (133). Doctors often worry about possible legal liability for potential harm to patients, which makes them cautious in recommending such drugs. Olson et al. also note concerns about financial toxicity in the use of medical cannabis drugs and highlight the need for financial subsidies to ensure equitable access to effective and safe therapy (134).

In addition, the use of non-standardized and homemade drugs is a problem that requires attention. Thus, a study of homemade cannabis products revealed significant concentrations of Δ9-THC and CBD in chocolate cakes soaked in cannabis, which highlights the problems of accurately measuring cannabinoid levels (135). Risks associated with such products include a high dose, which can lead to excessive consumption and adverse psychological effects.

The study has several limitations. First, we analyzed only nine cannabinoid-related hashtags on the Instagram platform. Secondly, Instagram users do not represent the entire population as a whole, and the studied data reflect the opinions of only those who actively use this social network. The analysis may not fully capture the perspectives of certain age groups, such as older individuals who are less likely to use social media, as well as those without internet access. These factors may limit the representativeness of our sample and affect the generalizability of the results. Social platforms like Facebook, Twitter, or YouTube can provide more information. For primary screening purposes, a limited sample size of posts was selected, allowing for a focus on the main topics and trends related to medical cannabinoid use and a qualitative analysis of available content. Studies of online discussions are time-dependent; in this study, data were collected as of a specific date. Future application of machine learning techniques may significantly enrich the analysis, increase predictive power of results, and facilitate deeper pharmacovigilance of specific products. These limitations highlight the importance of taking a broader and more comprehensive approach to analysis that includes additional hashtags, data from different sources, and spans different time spans.

## Conclusion

Analysis of Instagram content showed high relevance of cannabis and cannabinoid-related posts, where educational and personal posts show significant relevance and interest among users. Advertising and commercial posts occupy a significant share, which emphasizes the active promotion of cannabis in everyday life but also indicates the need for closer control over advertising practices. Multilingual content confirms international interest and the need to take into account cultural characteristics. Organizations publishing posts show a higher level of relevance and professionalism in content compared to individual users, making them important sources of information and promotion of the topic of cannabis.

Analysis of the posts showed that personal stories, especially in English and Turkish, receive high engagement and a significant number of “likes,” indicating a strong emotional connection between the audience and the content. Educational posts in English and Spanish highlight the importance of scientific information on cannabis and its use. Promotional content in German and Portuguese is also getting attention, while posts about legalization in South Korea get a high like rating in the “other” category. The main hashtags and keywords indicate a high interest in cannabidiol and tetrahydrocannabinol, their positive effects, and medical use.

Instagram discussions about the use of cannabinoids for the treatment of various diseases demonstrate consistency with scientific research data confirming their effectiveness and the manifestation of side effects. These posts highlight the successful use of cannabinoids to treat epilepsy, multiple sclerosis, cachexia, chronic pain, nausea, vomiting caused by chemotherapy, and dementia. Data from scientific studies confirm that there are enough positive results that indicate the effectiveness of cannabidiol, sativex, and dronabinol in improving the symptoms of these diseases in palliative medicine. Clinical studies show that some cannabinoids are most effective in combination with other drugs, especially when previous therapy has not yielded the desired result or has been ineffective. Despite the ambiguity and controversy of the use of cannabinoids, they are of significant scientific and practical interest in terms of their pharmacological activity. However, posts and scientific publications also highlight the need to consider side effects and interactions with other medications. For a more accurate assessment of their safety and the development of optimal dosages, it is necessary to continue clinical studies.

Advertising and commercial posts can contribute to an increase in cannabis use, which requires increased risk awareness and the introduction of preventive measures. In the “other” category, users are actively discussing the legalization of medical cannabis and patient accessibility, reflecting the need to address these issues at the legislative level. It is important to continue monitoring and researching the health effects of cannabinoids and to develop recommendations to reduce the negative effects of their use.

Overall, social media content analysis can complement traditional scientific research by providing more information about how cannabis is used and perceived in real life and help develop safer and more effective guidelines for its use.

## Data Availability

The original contributions presented in the study are included in the article/supplementary material, further inquiries can be directed to the corresponding authors.
